# Subregional Structural Alterations in Hippocampus and Nucleus Accumbens Correlate with the Clinical Impairment in Patients with Alzheimer’s Disease Clinical Spectrum: Parallel Combining Volume and Vertex-Based Approach

**DOI:** 10.3389/fneur.2017.00399

**Published:** 2017-08-15

**Authors:** Xiuling Nie, Yu Sun, Suiren Wan, Hui Zhao, Renyuan Liu, Xueping Li, Sichu Wu, Zuzana Nedelska, Jakub Hort, Zhao Qing, Yun Xu, Bing Zhang

**Affiliations:** ^1^State Key laboratory of Bioelectronics, School of Biological Sciences and Medical Engineering, Southeast University, Nanjing, China; ^2^Institute of Cancer and Genetic Science, University of Birmingham, Birmingham, United Kingdom; ^3^Department of Neurology, Affiliated Drum Tower Hospital of Nanjing University Medical School, Nanjing, China; ^4^Department of Radiology, Affiliated Drum Tower Hospital of Nanjing University Medical School, Nanjing, China; ^5^Department of Neurology, Memory Clinic, 2nd Faculty of Medicine, Charles University in Prague, Motol University Hospital, Prague, Czechia; ^6^International Clinical Research Center, St. Anne’s University Hospital Brno, Brno, Czech Republic

**Keywords:** Alzheimer’s disease, mild cognitive impairment, deep gray matter structures, surface alteration, vertex analysis

## Abstract

Deep gray matter structures are associated with memory and other important functions that are impaired in Alzheimer’s disease (AD) and mild cognitive impairment (MCI). However, systematic characterization of the subregional atrophy and deformations in these structures in AD and MCI still need more investigations. In this article, we combined complex volumetry- and vertex-based analysis to investigate the pattern of subregional structural alterations in deep gray matter structures and its association with global clinical scores in AD (*n* = 30) and MCI patients (*n* = 30), compared to normal controls (NCs, *n* = 30). Among all seven pairs of structures, the bilateral hippocampi and nucleus accumbens showed significant atrophy in AD compared with NCs (*p* < 0.05). But only the subregional atrophy in the dorsal–medial part of the left hippocampus, the ventral part of right hippocampus, and the left nucleus accumbens, the posterior part of the right nucleus accumbens correlated with the worse clinical scores of MMSE and MOCA (*p* < 0.05). Furthermore, the medial–ventral part of right thalamus significantly shrank and correlated with clinical scores without decreasing in its whole volume (*p* > 0.05). In conclusion, the atrophy of these four subregions in bilateral hippocampi and nucleus accumbens was associated with cognitive impairment of patients, which might be potential target regions of treatment in AD. The surface analysis could provide additional information to volume comparison in finding the early pathological progress in deep gray matter structures.

## Introduction

Alzheimer’s disease (AD) is the most prevalent form of age-related dementia (1, 2) and is usually preceded by a stage of cognitive decline. This clinical state of cognitive decline is conceptualized as mild cognitive impairment (MCI) (3, 4). Previous studies showed that MRI-based assessments of brain volume, such as hippocampal atrophy, can provided additional information for diagnosis of AD (5). Specifically deep gray matter structures, including bilateral nucleus accumbens (NAc), amygdala, caudate nucleus, hippocampus, pallidum, putamen, and thalamus, are associated with memory, emotional learning, shifting attention, and spatial working memory (6, 7), which were typically impaired in AD stage. Previous studies have focused on these regions and reported that there was atrophy on hippocampus and entorhinal cortex (8–12). Besides, it also suggested that other deep gray matter structures, like amygdala, putamen, and thalamus also showed significantly reduced volume, even in MCI (10, 13–15).

However, the subregions of deep gray matter structures may have different function and their impairment in AD may also have subregional specificity (9, 15–17). Therefore, it is very informative to explore the changes of deep gray matter structures on the subregional level in the AD and MCI. Previous studies have reported shape abnormalities in multiple deep gray matter structures, such as the dorsal–medial part of thalamus (15), the anterior hippocampus (9, 17), and the basolateral complex of amygdala (16, 17) in AD patients compared to controls. However, there is still lack of a systematic investigation of all deep gray matter structures.

In the current study, we unitized the automated software package, FMRIB’s Integrated Registration and Segmentation Tool (FIRST) (16, 18, 19), which provided a powerful tool to analyze subregional atrophy and shape alterations within seven pairs of deep gray matter structures. The volumes of each deep gray matter structure and the vertex-wised distortion were compared among clinically normal controls (NCs) as well as MCI and AD patients. We hypothesized that the pattern of atrophy on subregional level would differ among the three mentioned groups, and the specific pattern of more profound atrophy or shape alterations would correlate with poorer scores of global clinical scores.

## Materials and Methods

### Subjects

Ninety consecutive subjects were recruited from the memory clinic of Affiliated Drum Tower Hospital of Nanjing University Medical School. All subjects underwent a clinical evaluation using Mini-Mental State Examination (MMSE) (20) and Montreal Cognitive Assessment (MOCA) (21). Written consent was obtained from all subjects or their proxies after a detailed explanation of the study procedures, which was approved by the local Ethics Committee.

The MCI patients (*n* = 30) were diagnosed based on the following criteria (22): (a) memory complaint, preferably confirmed by an informant; (b) objective memory impairment, adjusted for age, and education; (c) normal or near-normal performance on general cognitive functioning and no or minimum impairment of daily life activities; and (d) not meeting the criteria for dementia. AD patients (*n* = 30) met NINCDS-ADRDA criteria (23). For comparison, we included NC (*n* = 30) who (a) had no cognitive complaints, (b) scored normally on global cognitive scales such as MMSE and MOCA, and (c) had no evidence of any structural abnormality on a conventional MRI scan.

Exclusion criteria were history of any significant medical, psychiatric, or neurological illness other than MCI or AD; history of brain injury; alcohol or drug abuse of alcohol; and missing clinical assessments.

### MRI Acquisition

Images were acquired on a 3.0-T MR scanner (Achieva 3.0T TX dual Medical Systems; Philips Medical Systems, Eindhoven, Netherlands) using a three-dimensional turbo fast echo (3D-TFE) T_1_-weighted pulse sequence with TR = 9.7 ms, TE = 4.6 ms, flip angle = 8°, slice thickness = 1 mm, slice gap = 1 mm, FOV = 256 mm × 256 mm, and matrix = 192.

### Image Processing

**Figure 1:**
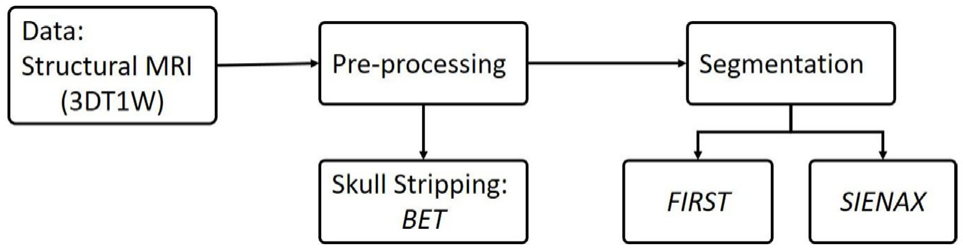
Flowchart of structural MR image processing.

The flowchart of image processing is shown in Figure 1. MRI volume were processed using FMRIB Software Library (FSL, Version 5.0, http://www.fmrib.ox.ac.uk/fsl) (18, 24). Briefly, brain extraction was performed on 3D T1-weighted MR images using Brain Extraction Tool (25), which uses a deformable model that evolves to fit the brain’s surface by the application of a set of locally adaptive model forces (25).

FMRIB’s Integrated Registration and Segmentation Tool (16) was applied to perform the segmentation and to measure volumes and vertexes in seven deep gray matter structures bilaterally, including NAc, amygdala, caudate nucleus, hippocampus, pallidum, putamen, and thalamus. FIRST initially performed a two-stage linear registration using 12 degrees of freedom, and the registration was performed by FLIRT (26). In the first stage, an affine registration of the whole-head to a standard space template (MNI template), with 1 mm × 1 mm × 1 mm resolution, was implemented. In the second stage, a subcortical mask was used to exclude regions outside the deep gray matter structures. Finally, a boundary correction was used to determine whether boundary voxels belong to this structure or not.

Based on this deep gray matter segmentation, the volume of each region was first extracted. The boundary of each region was reconstructed into a vertex-based surface, and the vertex locations from each subject (at a corresponding anatomical point) were projected onto the surface normal of the average shape of all the subjects. The projections were scalar values representing the signed, perpendicular distance from the average surface, where a positive value was outside the surface and a negative value was inside. Therefore, these scalar values of the vertex-based projection represented the shape information of each subjects and were used in the analysis of vertex-level group difference and to evaluate the shape deformation.

To reduce the influence of variation in head size, all volumes were scaled by normalized brain volume (NBV) estimated using SIENAX-FSL tool (24, 27). The segmentation result of each subject was visually checked and no error was observed. In the following statistical analysis, the volumes of each structure for each individual subject were corrected for NBV.

### Statistical Analysis

#### Volume-Based Analysis

After calculating the volumes, we used SPSS v 21.0 to analyze the data, including two statistical methods: analysis of covariance (ANCOVA) and linear regression. To correct the differences in head size among individual participants, volumes of each structure were scaled by NBV using the following equation:

$$V_{\text{standard}}=\left(V_{\text{roi}}\text{/}V_{\text{NBV}}\right)\times 10^{6}$$

where *V*_standard_ means the standardized volume corrected with NBV, *V*_roi_ means the absolute volume of each segmented structure, and *V*_NBV_ means NBV.

Analysis of covariance model was used to evaluate group-wise differences in volume measures between AD, MCI and NC, with controlling for age and gender. In this model, volumes of deep gray matter structures were included as dependent factors and diagnosis as independent factors. A correction for multiple comparisons was carried out using the false discovery rate (FDR) correction at *p* < 0.05. Subsequently, *post hoc* pair-wise comparisons were applied to identify between-group differences with *p*-value ≤0.05, which is considered as significant. Second, a linear regression model was applied to measure the associations between global clinical scores (MMSE and MOCA score) and volumes of each structure among all of the subjects. In the linear regression model, global clinical scores were included as the dependent variables and the volumes of deep gray matter structure were included as independent variables. Age, gender and NBV were included as covariates. A collinearity test was also performed to rule out multicollinearity between age and the volumes of deep gray matter structure.

#### Vertex-Based Analysis

To carry out the vertex analysis, we used FSL dedicated tools, generalized linear model (18) to design the statistical matrix and Randomize (28) to perform permutation inference. Two statistical models were also designed in the vertex analysis.

First, ANCOVA was applied to determine the subregional changes within the deep gray matter structures across AD, MCI, and NC groups. Second, we correlated local subregional changes with global clinical scores at *p*-value ≤0.05 across all subjects. Age and gender were taken into as covariates in both of the ANCOVA and correlational analyses. We applied the FDR (*q* = 0.05) to correct for the multiple comparisons. The MNI coordinates of the center of gravity (COG) (18) at subregional alterations were also calculated for a better awareness of the locations where subregional changes occurred.

## Results

### Group Characteristics

Demographic and clinical characteristics of three groups are listed in Table 1. The AD, MCI, and NC groups did not differ in NBV (*p* = 0.052) and gender (*p* = 0.194), but differ in age (*p* = 0.028). As expected, the pathological alteration among groups led to the significant difference in the scores of MMSE and MOCA among patients with AD, MCI, and NC (*p* < 0.001).

**Table 1:** Study sample characteristics.

Group	AD (*n* = 30)	MCI (*n* = 30)	NC (*n* = 30)	*p*-Value
Age (years; SD)	74.1 ± 10.5	74.6 ± 9.6	68.0 ± 11.0	0.028*
Gender (M/F)	13:17	17:13	20:10	0.194
NBV (cm^3^; SD)	1,154.6 ± 114.9	1,225.6 ± 116.6	1,183.5 ± 102.9	0.052
MMSE (SD)	16.5 ± 5.5	25.4 ± 2.1	29.0 ± 1.2	<0.001**
MOCA (SD)	11.9 ± 4.7	21.9 ± 2.1	26.9 ± 2.1	<0.001**

*p ≤ 0.05.

**p ≤ 0.01.

*Demographic data are presented as means ± SDs for continuous and proportions for categorical variables*.

*AD, Alzheimer’s disease; MCI, mild cognitive impairment; NC, normal controls; NBV, normalized brain volume; MMSE, Mini-Mental State Examination; MOCA, Montreal Cognitive Assessment; M, male; F, female*.

### Deep Gray Matter Structures Volumes in AD and MCI Groups

Group-wise differences in volumes of the deep gray matter structure are displayed in Table 2. Volumes of bilateral NAc and bilateral hippocampi (Hipp) were smaller in AD, compared to NC group (L_Hipp: *p* = 0.001, R_Hipp: *p* = 0.001, L_NAc: *p* = 0.003, R_NAc: *p* = 0.003). MCI had smaller left hippocampus (*p* = 0.020), right NAc (*p* = 0.010), and left putamen (*p* = 0.038) volumes, compared to the NC group. Only right hippocampus showed a smaller volume in AD, compared to MCI group (*p* = 0.012).

**Table 2:** Group-wise differences in the deep gray matter structures.

Structure	AD, mean ± SD	MCI, mean ± SD	NC, mean ± SD	*t*-Test (*p*-value)		
				AD-MCI	AD-NC	MCI-NC
L_NAc	316.5 ± 135.0	355.9 ± 126.2	443.3 ± 89.2	0.171	0.003**	0.078
R_ NAc	215.7 ± 104.5	228.1 ± 105.0	322.6 ± 83.2	0.585	0.003**	0.010**
L_Hipp	2,583.5 ± 591.0	2,724.7 ± 544.9	3,166.8 ± 374.9	0.283	0.001**	0.020*
R_Hipp	2,652.4 ± 625.7	2,988.9 ± 493.7	3,283.6 ± 386.3	0.012*	0.001**	0.240
L_Put	3,395.1 ± 804.1	3,409.9 ± 466.9	3,923.5 ± 618.7	0.853	0.062	0.038*
R_Put	3,415.8 ± 770.5	3,461.3 ± 591.0	3,847.9 ± 604.6	0.981	0.276	0.277
L_Thal	5,100.6 ± 650.5	5,033.1 ± 773.1	5,466.3 ± 534.6	0.592	0.352	0.145
R_Thal	4,833.0 ± 570.8	4,754.2 ± 687.0	5,152.1 ± 516.8	0.515	0.367	0.125

*p ≤ 0.05; **p ≤ 0.01 using ANCOVA; FDR corrected for t-test and adjusted for normalized brain volume, age, and gender.

*Significant across and between-group differences are in bold*.

*L, left; R, right; NAc, nucleus accumbens; Hipp, hippocampus; Put, putamen; Thal, thalamus*.

### Associations between Volumes and Clinical Rating Scales

We did not find a multicollinearity among the selected variables: age and volumes of deep gray matter structures. After controlling age, gender, and NBV, the *p*-values and β-regression coefficient of the correlations between volumes of deep gray matter structures and the global clinical scores across all subjects are displayed in Table 3. The volumes of bilateral NAc, bilateral hippocampi, which also showed significant atrophy in AD, compared to NC, were significantly correlated with MMSE and MOCA scores (*p* ≤ 0.05). The volumes of other deep gray matter structures did not show significant correlations with clinical scores (*p* > 0.05).

**Table 3:** Association between volumes in deep gray matter structures and global clinical scores.

Structure	MMSE	MOCA
L_NAc	0.359 (0.002**)	0.382 (0.001**)
R_NAc	0.277 (0.024*)	0.324 (0.007**)
L_Hipp	0.389 (0.001**)	0.359 (0.001**)
R_Hipp	0.383 (0.001**)	0.369 (0.001**)
L_Put	0.103 (0.377)	0.078 (0.493)
R_Put	0.174 (0.139)	0.139 (0.232)
L_Thal	0.074 (0.522)	0.008 (0.945)
R_Thal	0.016 (0.888)	0.000 (0.998)

*Results are presented as β-coefficient (*p*-values) based on linear regression model*.

*p ≤ 0.05.

**p ≤ 0.01.

### Vertex Analysis of Deep Gray Matter Structures

**Figure 2:**
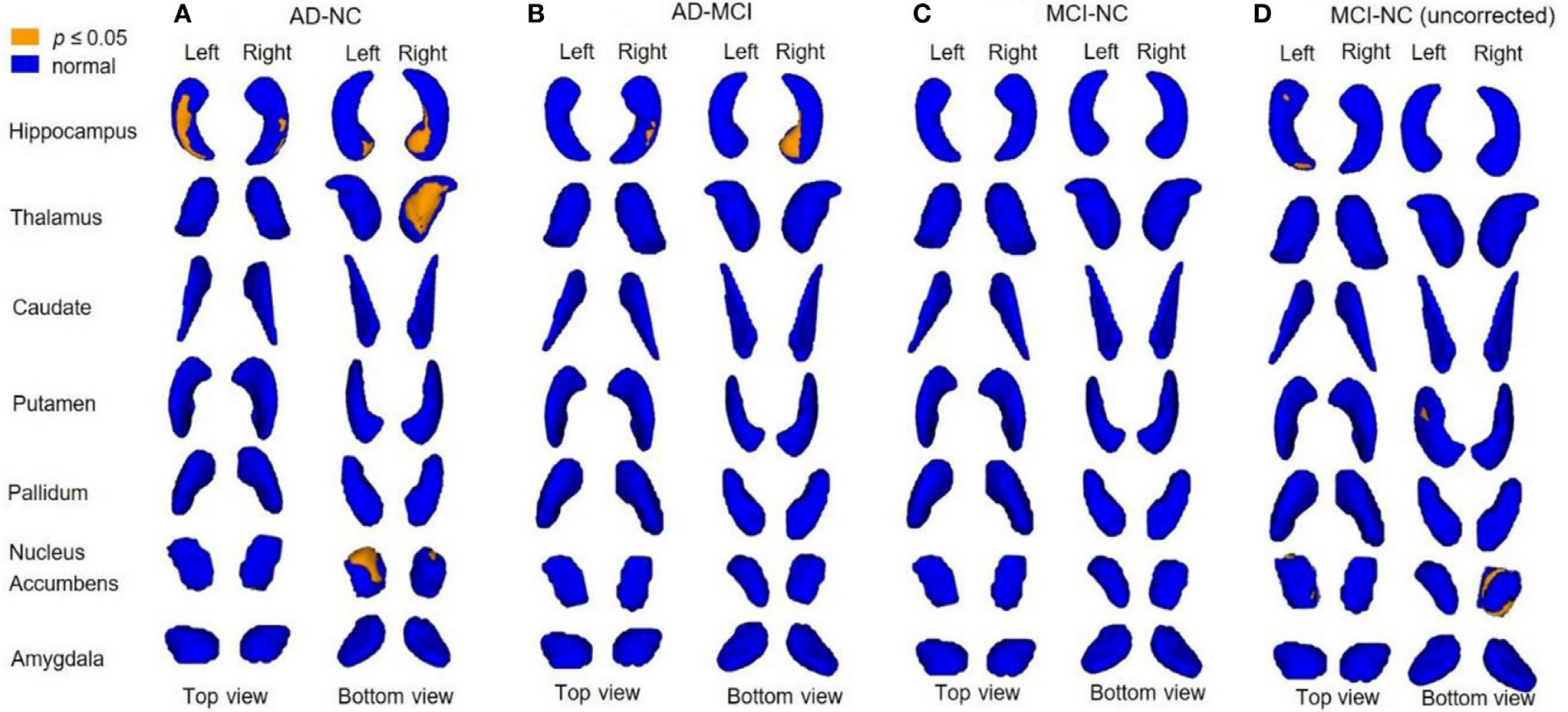
Analysis of covariance (ANCOVA) of between-group shape differences. 3D rendering of 1 − *p* value images with vertex analysis between-group comparisons showing the subregional changes (yellow color coded) of deep gray matter structures [adjusted for age and gender; *p*-value corrected for multiple comparisons using false discovery rate (FDR)]. Shape alterations are seen in Alzheimer’s disease (AD) compared to normal control (NC) **(A)** in bilateral hippocampi, bilateral nucleus accumbens (NAc), right thalamus; AD compared to mild cognitive impairment (MCI) **(B)** in right hippocampus; MCI showed no significant alteration compared to NC after FDR corrected **(C)**, while there are alterations found in left hippocampus, right NAc, and left putamen before FDR corrected **(D)**.

**Figure 3:**
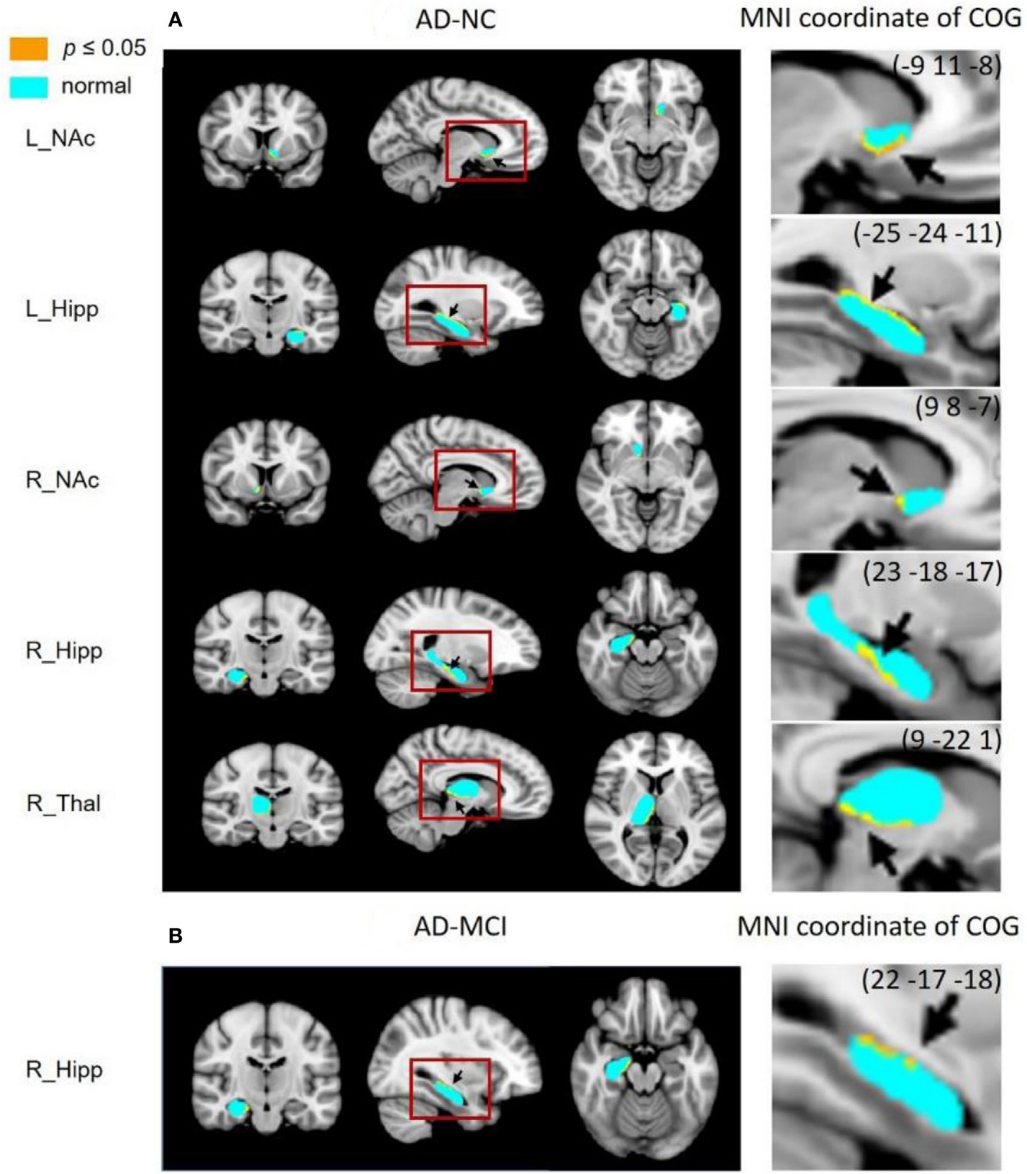
Subregional surface alterations in hippocampus, nucleus accumbens (NAc), and thalamus, indicating the MNI coordinates of subregions where shape changes occurred (marked with an arrow). COG, center of gravity. **(A)** AD-NC and **(B)** AD-MCI.

The results from vertex analysis are illustrated as probabilistic images that show the significant shape abnormalities within deep gray matter structures with 1 − *p* ≥ 0.95 (*p* ≤ 0.05) in Figure 2. After controlling for age and gender, the surface abnormalities were located within bilateral hippocampi and NAc in AD, compared to NC group, and only shape alterations in the right hippocampus were found in AD, compared to MCI group. Specifically, significant shape differences were detected in the ventral part of left NAc (left ventral NAc) and right hippocampus (right ventral hippocampus), posterior part of right NAc (right posterior NAc), the dorsal–medial part of left hippocampus (left dorsal–medial hippocampus), and medial–ventral part of right thalamus (right medial–ventral thalamus) in AD when compared to NC. Less surface alterations were found in left hippocampus, right NAc, and left putamen in MCI, compared to NC without correction for the multiple comparisons, while these alterations did not survive under FDR correction. Figure 3 shows the location of shape abnormalities using MNI coordinates of COG (colored in yellow).

### Relationship between Regional Shape Alterations and Clinical Measures

**Figure 4:**
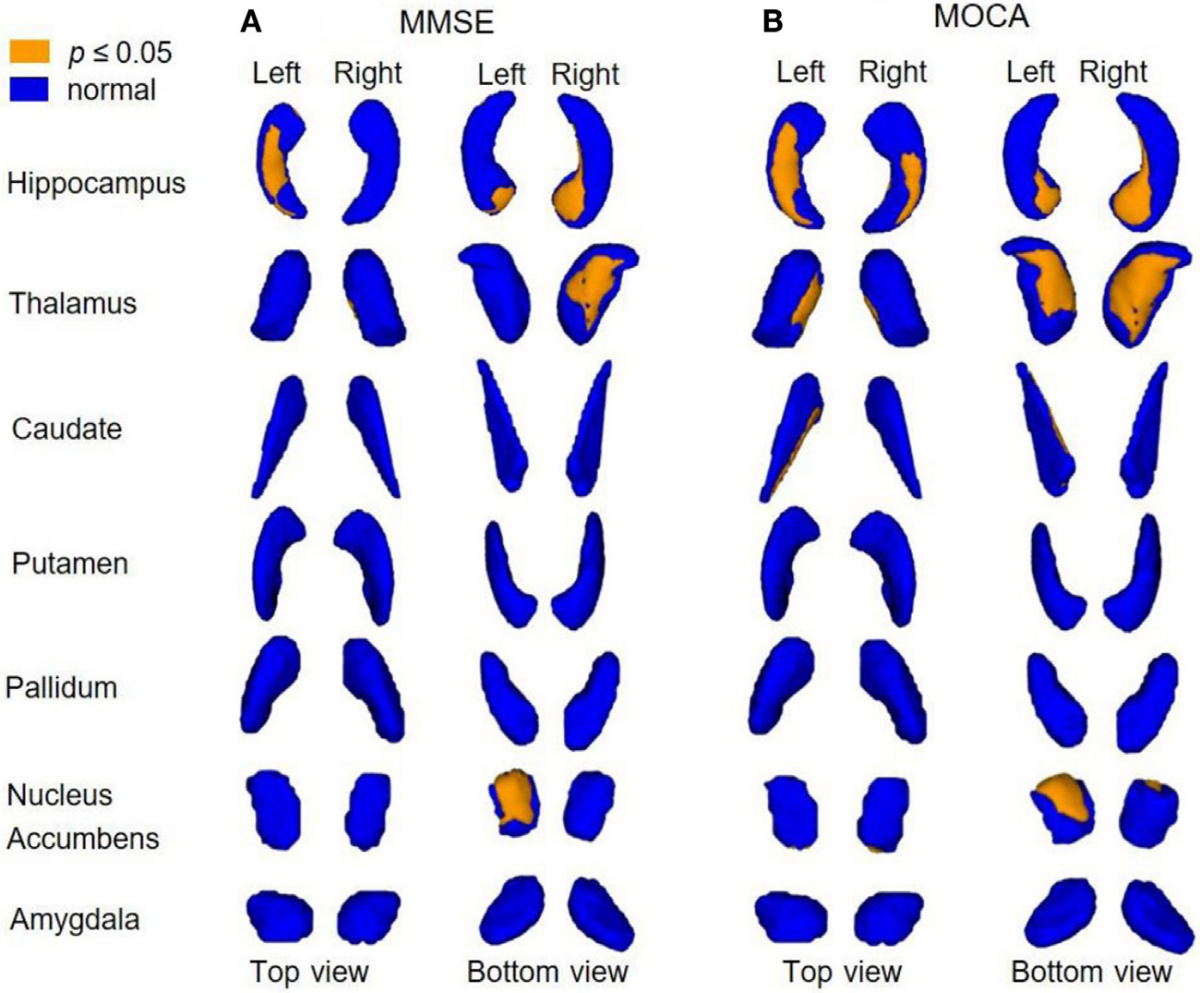
Correlations between subregional shape alterations and global clinical scores. This 3D rendering of 1 − *p* value images shows the correlations (yellow color coded) between subregional atrophy and clinical scores (adjusted for age and gender; FDR corrected). Across the whole data cohort, correlations were observed **(A)** in hippocampi, left NAc, and right thalamus and MMSE scores; **(B)** in hippocampi, NAc, and thalami and MOCA scores.

Figure 4 shows correlations between shape alterations of deep gray matter structures and clinical measures of MMSE and MOCA within the whole sample. MMSE scores were associated with shape abnormalities in bilateral hippocampi, left NAc, and right thalamus, whereas MOCA scores were associated with shape abnormalities in bilateral hippocampi, NAc, and thalami. The localization of subregional alterations and their correlation with MMSE and MOCA scores is shown in Table 4. It can be observed that the left dorsal–medial hippocampus, the right ventral hippocampus, the left ventral NAc, and the right medial–ventral thalamus significantly correlated with MMSE and MOCA, while the right posterior NAc and the medial–ventral part of the left thalamus only correlated with MOCA. Notably, compared with NC, these results are quite consistent with the atrophy regions found in AD groups, which are shown in Figures 2 and 3.

**Table 4:** Associations between subregional shape alterations and clinical measures (corresponding to the yellow areas in Figure 4).

Structure	L_NAc	R_NAc	L_Hipp	R_Hipp	L_Thal	R_Thal
MMSE	Ventral	–	Dorsal–medial	Ventral	–	Medial–ventral
MOCA	Ventral	Posterior	Dorsal–medial	Ventral	Medial–ventral	Medial–ventral

## Discussion

In this study, we assessed the pattern of subregional structural alterations in deep gray matter structures in AD and MCI patients. Compared with NC, we found that among all seven pairs of structures, the bilateral hippocampi and NAc showed significant atrophy in AD. But only the atrophy in the left dorsal-medial hippocampus, the right ventral hippocampus, the left ventral NAc and the right posterior NAc correlated with the worse global clinical scores. These results suggested that the atrophy of these four subregions in bilateral hippocampi and NAc was associated with clinical impairment in MCI and AD patients, which might be useful in diagnosis, evaluation and management of AD. Furthermore, compared with NC, the volume of bilateral thalami did not significantly decrease in AD patients, but the significant atrophy was observed in the right medial-ventral thalamus and correlated with global clinical scores by using surface analysis. These results implied that the surface analysis could provide additional information for volume comparison in finding the early pathological progress.

It is well known that hippocampus plays an important role in spatial, semantic, and episodic memory, which are generally impaired in AD and MCI patients (29–32). Numerous studies indicated that hippocampal atrophy contributes to memory impairment in patients with AD (33–38). Recent studies in AD found that obvious atrophy in hippocampal CA1, especially in MCI patients who converted to AD over time (37, 38). In the current study, we found that hippocampal atrophy and its correlation with global clinical scores. Moreover, vertex analysis showed a alteration in the right ventral hippocampus and the left dorsal-medial hippocampus. This result is in line with a previous report that hippocampal atrophy is not homogeneous across the various hippocampal subregions during the progression of AD (5, 39). The dorsal hippocampus has been studied extensively for its significant role in spatial working memory, especially when processing the short-term spatial memory (40). Fanselow and Dong reported that the dorsal hippocampus performs primarily cognitive functions, while the ventral hippocampus relates to stress, emotion, and affect (41). Other studies also indicated that loss of neurogenesis in the dorsal and ventral hippocampus was associated with the impaired cognitive function (42), and the ventral hippocampus supported working memory for odor information (43). Experiments in rats showed that dorsal-medial hippocampus plays a role in flexible, adaptive behavior, and cognitive processing, and rats with dorsal-medial hippocampus lesions suffered significant working memory deficits (44). In line with the previous studies, the significant correlation between the dorsal/ventral hippocampal shape distortion and the global clinical scores in our study also emphasized the critical role of these two hippocampal subregions in cognitive impairment in AD process. Besides, the subregional level atrophy in hippocampi, which is slightly in MCI patients, but dramatically deteriorated in AD, further supporting the concept that AD is generally converted from MCI. Therefore, treatment on dorsal/ventral hippocampus may be effective to prevent the progression of AD. Drug treatment upon reduction of memory consolidation in rats with impaired dorsal hippocampus indicated that morphine may prevent impairment of memory consolidation (45). Yiu et al. also reported that targeting CREB in the CA1 region of dorsal hippocampus may be a useful therapeutic strategy in treating humans with AD (46). The role of the ventral hippocampus in AD is still not clear. Since the stress management related to the ventral hippocampus is suggested as a useful intervention in early AD and MCI (47), the treatment on ventral hippocampus for AD still need to be explored. The measurement in hippocampal subregion might be useful in monitoring the effect of treatment or intervention on subregional level.

In addition to hippocampus, NAc participates in integrating the information involved in learning and executive function and is also related to the cognitive processing of aversion, motivation, pleasure, reward, and reinforcement learning (48, 49). NAc is part of the striatum and has close connections with both limbic structures of the hippocampus, the amygdala, and the prefrontal cortex. One previous research about late-onset Alzheimer’s disease patients showed the significant reductions in NAc volumes (50). Some previous studies have shown that atrophy of NAc occurred in patients with AD (51, 52), while they failed to reveal its function in cognitive decline. In our study, our results were on par with these previous studies that the bilateral NAc were more atrophied in AD and MCI patients compared to NCs. Furthermore, we found the atrophy in NAc was correlated with MMSE and MOCA scores. These results suggested that in addition to hippocampus, the atrophy in bilateral NAc may also play an important role in clinical impairment in AD and MCI. The localized atrophy in NAc has been confirmed to be associated with apathy in Parkinson’s disease, and the severity of apathy was correlated with morphological changes in this region (53). Comparably, the vertex analyses in our study found that the atrophy of NAc and its association with clinical scores were both constrained within the ventral and posterior aspects in AD. Our results suggested that the ventral/posterior parts of NAc may involve in AD progression. However, few studies have explored the function of NAc in subregional level, especially its functions in AD or MCI, which need to be investigated in the future. A previous study reported that the deep brain stimulation of NAc could be a new treatment strategies to addiction (54). In the future, treatment like deep brain stimulation on subregional level of NAc may be considered for intervention in AD patients.

Some studies have revealed that other deep gray matter structures, such as amygdala, basal nuclei, and thalamus, also suffer atrophy in AD patients (55). Shape changes of ventricle system were reported in ventricular regions adjacent to amygdala, caudate nucleus, and thalamus in AD patients (56). MRI study showed strongly reduced volumes of putamen and thalamus in AD patients and this atrophy may contribute to cognitive impairment (10). Volumes of caudate and thalamus in familial AD reduced at a presymptomatic stage (57). It is well recognized that thalamus is essential for generating attention (58) and is involved as a subcortical hub in many different neuronal pathways related to emotional, motivational and cognitive abilities that are impaired in AD (6). To our knowledge, few studies have investigated the subregional atrophy and its relationship with global clinical scores in thalami. Significant bilateral volumetric and subregional atrophy in the dorsal–medial part of the thalamus in AD patients has already been reported (10, 15). Another study found the subregional atrophy in the medial part of bilateral thalami (59), but not explored the relationship with clinical scores. Our study revealed significant shape abnormalities in the right medial-ventral thalamus in AD patients, but not found smaller volumes in bilateral thalami. Furthermore, the atrophy in right medial-ventral thalamus significantly correlated with MMSE and MOCA, suggesting that some parts of thalamus may be more vulnerable in AD and MCI. Our results suggest a specific atrophy pattern of the thalamus in AD and MCI, and also indicated that vertex-based (shape) analysis may provide additional and valuable information to popular volumetric analysis.

The main limitation of our study is that, although we adjusted our analysis for age, gender, and NBV, relatively small group samples may have reduced the statistical power. Therefore, some counterintuitive results were also need to be further validated in the future. For example, the putamen volume was significantly different between MCI and NC, but not between AD and NC. Furthermore, this was a cross-sectional study, and so it was difficult to judge whether atrophy of specific deep gray matter structures was a primary or secondary phenomenon to the hippocampal or entorhinal cortex. Therefore, further and preferable longitudinal investigations are needed to confirm the relationship between clinical and cognitive decline and the subregional atrophy patterns.

## Conclusion

Based both on volumetric and vertex-based (shape) analysis of deep gray matter structures, this study showed that atrophy of deep gray matter structures in AD and MCI patients occurs at subregional level even when the volume of the whole given structure is not reduced. And this subregional atrophy within hippocampi and NAc correlates with worse global clinical scores. Atrophy measurement on subregional level may be useful in early diagnosis and management of AD patients or in evaluation of the treatment strategies.

## Author Contributions

XLN, YS, and SRW contributed to this article equally, being responsible for the image procession, data analysis, and writing the article. HZ, RYL, XPL, and SCW are responsible for the MRI acquisition and data collection; ZN, JH, ZQ, and YX give advice on how to do the data analysis and modify the article. BZ put forward the research ideas and guide the whole study.

## Conflict of Interest Statement

The authors declare that the research was conducted in the absence of any commercial or financial relationships that could be construed as a potential conflict of interest.

## References

[1] Schott J Kennedy J Fox N. New developments in mild cognitive impairment and Alzheimer’s disease. Curr Opin Neurol (2006) 19:552–8. 10.1097/01.wco.0000247611.44106.76 17102693

[2] Yaari R Corey-Bloom J. Alzheimer’s disease. Semin Neurol (2007) 27:32–41. 10.1055/s-2006-956753 17226739

[3] Petersen RC Doody R Kurz A Mohs R Morris J Rabins P Current concepts in mild cognitive impairment. Arch Neurol (2001) 58:1985–92. 10.1001/archneur.58.12.1985 11735772

[4] Peterson RC. Mild cognitive impairment: current research and clinical implications. Semin Neurol (2007) 27:22–31. 10.1055/s-2006-956752 17226738

[5] Carlesimo GA Piras F Orfei MD Iorio M Caltagirone C Spalletta G Atrophy of presubiculum and subiculum is the earliest hippocampal anatomical marker of Alzheimer’s disease. Alzheimers Dement (2015) 1:24–32. 10.1016/j.dadm.2014.12.001 PMC4876901 27239489

[6] Herrero MT Barcia C Navarro JM Functional anatomy of thalamus and basal ganglia. Childs Nerv Syst (2002) 18:386–404. 10.1007/s00381-002-0604-1 12192499

[7] Rugg MD Yonelinas AP. Human recognition memory: a cognitive neuroscience perspective. Trends Cogn Sci (2003) 7:313–9. 10.1016/S1364-6613(03)00131-1 12860190

[8] Fox NC Freeboroug PA. Brain atrophy progression measured from registered serial MRI: validation and application to Alzheimer’s disease. J Magn Reson Imaging (1997) 7:1069–75. 10.1002/jmri.1880070620 9400851

[9] Gerardin E Chételat G Chupin M Cuingnet R Desgranges B Kim H Multidimensional classification of hippocampal shape features discriminates Alzheimer’s disease and mild cognitive impairment from normal aging. Neuroimage (2009) 47:1476–86. 10.1016/j.neuroimage.2009.05.036 19463957 PMC3001345

[10] de Jong LW van der Hiele K Veer IM Houwing JJ Westendorp RG Bollen EL Strongly reduced volumes of putamen and thalamus in Alzheimer’s disease: an MRI study. Brain (2008) 131:3277–85. 10.1093/brain/awn278 19022861 PMC2639208

[11] Scheltens P Leys D Barkhof F Huglo D Weinstein H Vermersch P Atrophy of medial temporal lobes on MRI in “probable” Alzheimer’s disease and normal ageing: diagnostic value and neuropsychological correlates. J Neurol Neurosurg Psychiatry (1992) 55:967–72. 10.1136/jnnp.55.10.967 1431963 PMC1015202

[12] Stoub TR Bulgakova M Leurqans S Bennett DA Fleischman D Turner DA MRI predictors of risk of incident Alzheimer disease: a longitudinal study. Neurology (2005) 64:1520–4. 10.1212/01.WNL.0000160089.43264.1A 15883311

[13] Alves GS O’Dwyer L Jurcoane A Oertel-Knöchel V Knöchel C Prvulovic D Different patterns of white matter degeneration using multiple diffusion indices and volumetric data in mild cognitive impairment and Alzheimer patients. PLoS One (2012) 7:e52859 10.1371/journal.pone.0052859 23300797 PMC3534120

[14] Cheng WC Cheng PE Liou M Group factor analysis for Alzheimer’s disease. Comput Math Methods Med (2013) 2013:1–8. 10.1155/2013/830237 PMC3603153 23533539

[15] Zarei M Patenaude B Damoiseaux J Morgese C Smith S Matthews PM Combining shape and connectivity analysis: an MRI study of thalamic degeneration in Alzheimer’s disease. Neuroimage (2010) 49:1–8. 10.1016/j.neuroimage.2009.09.001 19744568

[16] Patenaude B Smith SM Kennedy DN Jenkinson M. A Bayesian model of shape and appearance for subcortical brain segmentation. Neuroimage (2011) 56:907–22. 10.1016/j.neuroimage.2011.02.046 21352927 PMC3417233

[17] Qiu A Fennema-Notestine C Dale AM Miller MI. Regional shape abnormalities in mild cognitive impairment and Alzheimer’s disease. Neuroimage (2009) 45:656–61. 10.1016/j.neuroimage.2009.01.013 19280688 PMC2847795

[18] Jenkinson M Beckmann CF Behrens TEJ Woolrich MW Smith SM. FSL. Neuroimage (2012) 62:782–90. 10.1016/j.neuroimage.2011.09.015 21979382

[19] Nugent AC Luckenbaugh DA Wood SE Bogers W Zarate CA Drevets WC. Automated subcortical segmentation using FIRST: test-retest reliability, interscanner reliability, and comparison to manual segmentation. Hum Brain Mapp (2013) 34:2313–29. 10.1002/hbm.22068 22815187 PMC3479333

[20] Folstein MF Folstein SE McHugh PR Mini-mental state: a practical method for grading the cognitive state of patients for the clinician. J Psychiatr Res (1975) 12:189–98. 10.1016/0022-3956(75)90026-6 1202204

[21] Smith T Gildeh N Holmes C The Montreal Cognitive Assessment: validity and utility in a memory clinic setting. Can J Psychiatry (2007) 52:329–32. 10.1177/070674370705200508 17542384

[22] Winblad B Palmer K Kivipelto M Jelic V Fratiglioni L Wahlund LO Mild cognitive impairment-beyond controversies, towards a consensus: report of the International Working Group on mild cognitive impairment. J Intern Med (2004) 256:240–6. 10.1111/j.1365-2796.2004.01380.x 15324367

[23] McKhann G Drachman D Folstein M Katzman R Price D Stadlan EM Clinical diagnosis of Alzheimer’s disease: report of the NINCDS–ADRDA work group under the auspices of department of health and human services task force on Alzheimer’s disease. Neurology (1984) 34:939–44. 10.1212/WNL.34.7.939 6610841

[24] Smith SM Jenkinson M Woolrich MW Beckmann CF Behrens TEJ Johansen-Berg H Advances in functional and structural MR image analysis and implementation as FSL. Neuroimage (2004) 23:S208–19. 10.1016/j.neuroimage.2004.07.051 15501092

[25] Smith SM. Fast robust automated brain extraction. Hum Brain Mapp (2002) 17:143–55. 10.1002/hbm.10062 12391568 PMC6871816

[26] Jenkinson MBP Brady M Smith S. Improved optimization for the robust and accurate linear registration and motion correction of brain images. Neuroimage (2002) 17:825–41. 10.1006/nimg.2002.1132 12377157

[27] Smith SM Zhang Y Jenkinson M Chen J. Accurate, robust and automated longitudinal and cross-sectional brain change analysis. Neuroimage (2002) 17:479–89. 10.1006/nimg.2002.1040 12482100

[28] Winkler AM Ridgway GR Webster MA Smith SM Nichols TE. Permutation inference for the general linear model. Neuroimage (2014) 92:381–97. 10.1016/j.neuroimage.2014.01.060 24530839 PMC4010955

[29] Cohen NJ Eichenbaum H Memory, Amnesia, and the Hippocampal System. Massachusetts: MIT Press (1993).

[30] Squire LR Schacter DL The Neuropsychology of Memory. New York: Guilford Press (2002).

[31] Mavrogiorgou P Gertz HJ Ferszt R Wolf R Bär KJ Juckel G Are routine methods good enough to stain senile plaques and neurofibrillary tangles in different brain regions of demented patients. Psychiatr Danub (2011) 23:334–9. 22075733

[32] Wood R Chan D The hippocampus, spatial memory and Alzheimer’s disease. ACNR (2015) 15:5–7.

[33] Dubois B Feldman HH Jacova C Dekosky ST Barberger-Gateau P Cummings J Research criteria for the diagnosis of Alzheimer’s disease: revising the NINCDS-ADRDA criteria. Lancet Neurol (2007) 6:734–46. 10.1016/S1474-4422(07)70178-3 17616482

[34] Chetelat G Baron JC. Early diagnosis of Alzheimer’s disease: contribution of structural neuroimaging. Neuroimage (2003) 18:525–41. 10.1016/S1053-8119(02)00026-5 12595205

[35] de Leon MJ Convit A DeSanti S Golomb J Tarshish C Rusinek H The hippocampus in aging and Alzheimer’s disease. Neuroimag Clin N Am (1995) 5:1–17. 7743078

[36] Laakso MP Soininen H Partanen K Helkala EL Hartikainen P Vainio P Volumes of hippocampus, amygdala and frontal lobes in the MRI-based diagnosis of early Alzheimer’s disease: correlation with memory functions. J Neural Transm Park Dis Dement Sect (1995) 9:73–86. 10.1007/BF02252964 7605591

[37] Tang X Holland D Dale AM Younes L MI M. The diffeomorphometry of regional shape change rates and its relevance to cognitive deterioration in mild cognitive impairment and Alzheimer’s disease. Hum Brain Mapp (2015) 36:2093–117. 10.1002/hbm.22758 25644981 PMC4474005

[38] Tang X Holland D Dale AM Younes L Miller M. Shape abnormalities of subcortical and ventricular structures in mild cognitive impairment and Alzheimer’s disease: detecting, quantifying, and predicting. Hum Brain Mapp (2014) 35:3701–25. 10.1002/hbm.22431 24443091 PMC4474087

[39] Wachinger C Salat DH Weiner M Reuter M. Whole-brain analysis reveals increased neuroanatomical asymmetries in dementia for hippocampus and amygdala. Brain (2016) 139:3253–66. 10.1093/brain/aww243 27913407 PMC5840883

[40] Lee I Kesner RP Time-dependent relationship between the dorsal hippocampus and the prefrontal cortex in spatial memory. J Neurosci (2003) 23:1517–23. 12598640 10.1523/JNEUROSCI.23-04-01517.2003 PMC6742248

[41] Fanselow MS Dong HW Are the dorsal and ventral hippocampus functionally distinct structures. Neuron (2010) 65:7–19. 10.1016/j.neuron.2009.11.031 20152109 PMC2822727

[42] Vetreno RP Crews FT Binge ethanol exposure during adolescence leads to a persistent loss of neurogenesis in the dorsal and ventral hippocampus that is associated with impaired adult cognitive functioning. Front Neurosci (2015) 9:35 10.3389/fnins.2015.00035 25729346 PMC4325907

[43] Kesner R Hunsaker M Ziegler W. The role of the dorsal and ventral hippocampus in olfactory working memory. Neurobiol Learn Mem (2011) 96:361–6. 10.1016/j.nlm.2011.06.011 21742047

[44] Sahina H Lesions of the Dorsal Medial Hippocampus Induce Different Forms of Repetitive Behaviour in the Rat. New Zealand: University of Canterbury (2015).

[45] Zarrindast MR Navaeian M Nasehi M. Influence of three-day morphine-treatment upon impairment of memory consolidation induced by cannabinoid infused into the dorsal hippocampus in rats. Neurosci Res (2011) 69:51–9. 10.1016/j.neures.2010.09.007 20888871

[46] Yiu AP Rashid AJ Josselyn SA Increasing CREB function in the CA1 region of dorsal hippocampus rescues the spatial memory deficits in a mouse model of Alzheimer’s disease. Neuropsychopharmacology (2011) 36:2169–86. 10.1038/npp.2011.107 21734652 PMC3176558

[47] Lombardo NBE Wu B Volicer L Martin A Serper LL Zhang XW Evidence based nutrition, exercise, cognitive rehabilitation & stress management interventions for Alzheimer’s disease: treatment or prevention? Neurobiol Aging (2004) 25:S206–7. 10.1016/S0197-4580(04)80692-4

[48] Nestler E Hyman S Malenka R Molecular Neuropharmacology: A Foundation for Clinical Neuroscience. 2nd ed New York, NY: McGraw-Hill Professional (2015).

[49] Wenzel JM Rauscher NA Cheer JF Oleson EB. A role for phasic dopamine release within the nucleus accumbens in encoding aversion: a review of the neurochemical literature. ACS Chem Neurosci (2015) 6:16–26. 10.1021/cn500255p 25491156 PMC5820768

[50] Pievani M Bocchetta M Boccardi M Cavedo E Bonetti M Thompson PM Striatal morphology in early-onset and late-onset Alzheimer’s disease: a preliminary study. Neurobiol Aging (2013) 34:1728–39. 10.1016/j.neurobiolaging.2013.01.016 23428181

[51] Hilal S Amin SM Venketasubramanian N Niessen WJ Vrooman H Wong TY Subcortical atrophy in cognitive impairment and dementia. J Alzheimer Dis (2015) 48:813–23. 10.3233/JAD-150473 26402115

[52] Möller C Dieleman N van der Flier WM Versteeg A Pijnenburg Y Scheltens P More atrophy of deep gray matter structures in frontotemporal dementia compared to Alzheimer’s disease. J Alzheimers Dis (2015) 44(2):635–47. 10.3233/JAD-141230 25322925

[53] Carriere N Besson P Dujardin K Duhamel A Defebvre L Delmaire C Apathy in Parkinson’s disease is associated with nucleus accumbens atrophy: a magnetic resonance imaging shape analysis. Mov Disord (2014) 29:897–903. 10.1002/mds.25904 24817690

[54] de Leon MJ George AE Stylopoulos LA Smith G Miller DC Early marker for Alzheimer’s disease: the atrophic hippocampus. Lancet (1989) 2:672–3. 10.1016/S0140-6736(89)90911-2 2570916

[55] Cherubini A Péran P Spoletini I Di Paola M Di Iulio F Hagberg GE Combined volumetry and DTI in subcortical structures of mild cognitive impairment and Alzheimer’s disease patients. J Alzheimers Dis (2010) 19:1273–82. 10.3233/JAD-2010-091186 20308792

[56] Ferrarini L Palm WM Olofsen H van Buchem MA Reiber JH Admiraal-Behloul F. Shape differences of the brain ventricles in Alzheimer’s disease. Neuroimage (2006) 32:1060–9. 10.1016/j.neuroimage.2006.05.048 16839779

[57] Ryan NS Keihaninejad S Shakespeare TJ Lehmann M Crutch SJ Malone IB Magnetic resonance imaging evidence for presymptomatic change in thalamus and caudate in familial Alzheimer’s disease. Brain (2013) 136:1399–414. 10.1093/brain/awt065 23539189 PMC3634199

[58] Newman J. Thalamic contributions to attention and consciousness. Conscious Cogn (1995) 4:172–93. 10.1006/ccog.1995.1024 8521257

[59] Cho H Kim J Kim C Ye B Kim H Yoon C Shape changes of the basal ganglia and thalamus in Alzheimer’s disease: a three-year longitudinal study. J Alzheimers Dis (2014) 40:285–95. 10.3233/JAD-132072 24413620

